# The Radical Cure of Femoral Hernia

**Published:** 1897-09-25

**Authors:** Alexander Miles

**Affiliations:** Surgeon to Leith Hospital


### THE RADICAL CURE OF FEMORAL HERNIA.

By ALEXANDER MILES, M.D., F.R.C.S.Edin., Surgeon to Leith Hospital.

Operations for the radical cure of femoral hernia are performed much less frequently, in proportion to the number of sufferers, than similar procedures for inguinal hernia; and this in face of the fact that the wearing of a truss is seldom, if ever, more than a palliative measure in the former variety of rupture. The reasons given for this are not entirely satisfactory. That the majority of patients being females are less exposed to such severe and sudden exertions as usually determine strangulation is only true of the non-labouring classes. Women who have to perform manual labour while suffering from femoral hernia run great risks, especially when it is borne in mind that strangulation when it occurs in this variety of hernia is more acute, and places the life of the patient in more imminent danger, than the same condition in inguinal hernia. If an operation offers hope of removing this risk, women, especially young working women, are entitled to the benefit of it. It has also been said that the more sedentary or less athletic habits of women, and the particular arrangement of their clothing, renders the wearing of a truss less irksome than in men, and so removes one of the reasons for operation. Many patients, however, do complain bitterly of the annoyance and discomfort of trusses, and in many cases the fact that the truss is necessary prevents the patient taking that amount of exercise which is essential to good health. Amongst the poorer classes the expense of keeping up trusses is a considerable item. Another fact which limits the number of operations for this condition is that most of those who suffer from femoral hernia are past the period of active labour and exercise. They are still, however, liable to all the risks which attend strangulation.

Doubtless the comparative want of success which followed the earlier operations for femoral, as contrasted with inguinal, hernia, has deterred many surgeons from attempting the radical cure, except in cases where herniotomy was being performed for strangulation. The older statistics seem to show that most of the operations were done under these circumstances, and, in spite of the unfavourable conditions, the results were so good that it is matter for wonder that the operation was not more frequently advised for uncomplicated cases. Wood operated on twenty cases, fourteen of which were strangulated and three irreducible, with only one death and one failure. Bank's results were also satisfactory, both as to mortality and issue. At the present time the operation is more frequently performed than the statements in most text-books would lead one to suppose, and certainly with greater success. An American surgeon, writing so recently as 1893, says "relapse is almost invariable, regardless of how the condition is treated by radical measures." This has not been the experience of British surgeons, either in the early days of the operation or more recently.

The essentials to success in this operation are—first, the complete obliteration of the hernial sac in such a manner as to leave no depression or even dimple in the region where the protrusion escaped. Some surgeons lay great stress on the importance of having the peritoneum in this region very tightly stretched; and Bennett points out that something may be gained in this direction by twisting the freed sac once or twice before obliterating it by ligature or otherwise. Others depend on plugs or buttresses, made from the sac, for strengthening the weak spot.

The second point aimed at is the closure of the canal through which the hernia passes. It is here that the technical difficulty lies in femoral hernia. The excessively tight, fibrous structures which bound the short crural canal prevent apposition of its walls; and the close proximity of the femoral vein renders it impossible to insert deep stitches without risk of puncturing, or of tightening shallower ones without risk of occluding it. Mr. Watson Cheyne has devised a plan which goes far towards solving the difficulty of how to close the canal. He dissects up a U-shaped flap from the substance of the pectineus muscle, and, folding it up, fixes it into the canal, where, after the muscular fibres have atrophied, it remains as a fibrous plug. The only objection to this procedure, as Mr. Bennett points out, is that the plug is inserted at the wrong end of the canal. A similar device, applicable to the abdominal end of the passage, would meet the case.

The operation of Kocher, of Berne, aims at not only closing the sac and tightening up the peritoneum in the region of the crural ring, but also at so altering the axis of the sac as to direct pressure away from the weak spot. As regards closing the canal, he contents himself with stitching together the fascial structures of the region as far as possible. The writer has performed this operation in seven or eight cases during the last year and a half, with results which, so far as the time which has elapsed enables one to judge, are satisfactory. The following case may serve as an instance:—

D. McO., aged 65, a small, thin man, was admitted to Leith Hospital suffering from a bruised back, the result of his having been thrown from a high van which capsized on the street. For 15 years he had suffered from a small reducible femoral hernia, which had, however, never given him any serious trouble. A few days after the injuries to his back enabled him to be out of bed, he found that his hernia tended to come down oftener, was more difficult to reduce, and caused him some pain. On August 8th, 1896, abdominal pain, sickness and vomiting, hiccough, and constipation which had lasted for two days, although flatus was passed, led to an examination of his hernia, which was found to be tender, tense, and irreducible. The same afternoon Kocher's operation[1] was performed under chloroform anæsthesia. An incision along the inner third of Poupart's ligament exposed a small, tense, thick-walled sac, which on being opened was found to contain a knuckle of darkly-congested bowel, adherent to the sac, and firmly constricted by Gimbernat's ligament. The constriction being relieved and the adhesions separated, the bowel was returned, and the sac freed up as far as possible inside the crural ring. A small cut was then made through that part of the external oblique which forms the outer pillar of the external abdominal ring, and a pair of forceps passed through this slit under the aponeurosis till the point appeared below Poupart's ligament. The fundus of the sac, being grasped by the forceps and pulled back under the aponeurosis and out through the slit, was fixed in position by a few catgut stitches embracing it and the boundaries of the crural ring. The wound was closed without drainage, and healed by first intention under two dressings. He was kept in bed for six weeks, and then got up, wearing a pad of wool and elastic bandage for four weeks longer. He then returned to work, without truss or bandage, and has had no further trouble from his hernia.

[1] For a detailed description of this operation, see Kocher's "Operative Surgery," translated by Stiles, 1895.

